# Reproducibility efforts as a teaching tool: A pilot study

**DOI:** 10.1371/journal.pcbi.1010615

**Published:** 2022-11-10

**Authors:** Nestoras Karathanasis, Daniel Hwang, Vibol Heng, Rimal Abhimannyu, Phillip Slogoff-Sevilla, Gina Buchel, Victoria Frisbie, Peiyao Li, Dafni Kryoneriti, Isidore Rigoutsos

**Affiliations:** 1 Computational Medicine Center, Thomas Jefferson University, Pennsylvania, United States of America; 2 Department of Neurology, Thomas Jefferson University, Pennsylvania, United States of America; 3 Neuroscience Department, Thomas Jefferson University, Pennsylvania, United States of America; 4 Department of Biochemistry and Molecular Biology, Thomas Jefferson University, Pennsylvania, United States of America; 5 Cancer Biology Department, Sidney Kimmel Cancer Center, Thomas Jefferson University, Pennsylvania, United States of America; 6 Independent Researcher, Hamburg, Germany; McGill University, CANADA

## Abstract

The “replication crisis” is a methodological problem in which many scientific research findings have been difficult or impossible to replicate. Because the reproducibility of empirical results is an essential aspect of the scientific method, such failures endanger the credibility of theories based on them and possibly significant portions of scientific knowledge. An instance of the replication crisis, analytic replication, pertains to reproducing published results through computational reanalysis of the authors’ original data. However, direct replications are costly, time-consuming, and unrewarded in today’s publishing standards. We propose that bioinformatics and computational biology students replicate recent discoveries as part of their curriculum. Considering the above, we performed a pilot study in one of the graduate-level courses we developed and taught at our University. The course is entitled Intro to R Programming and is meant for students in our Master’s and PhD programs who have little to no programming skills. As the course emphasized real-world data analysis, we thought it would be an appropriate setting to carry out this study. The primary objective was to expose the students to real biological data analysis problems. These include locating and downloading the needed datasets, understanding any underlying conventions and annotations, understanding the analytical methods, and regenerating multiple graphs from their assigned article. The secondary goal was to determine whether the assigned articles contained sufficient information for a graduate-level student to replicate its figures. Overall, the students successfully reproduced 39% of the figures. The main obstacles were the need for more advanced programming skills and the incomplete documentation of the applied methods. Students were engaged, enthusiastic, and focused throughout the semester. We believe that this teaching approach will allow students to make fundamental scientific contributions under appropriate supervision. It will teach them about the scientific process, the importance of reporting standards, and the importance of openness.

## Author summary

Many scientific findings have been difficult or impossible to reproduce, resulting in the “replication crisis.” Because the repeatability of empirical data is a vital component of the scientific process, such errors imperil the validity of hypotheses dependent on them and potentially significant amounts of scientific knowledge. Nevertheless, direct replications are expensive, time-consuming, and unrewarded in today’s publishing standards. Thus, we suggest that bioinformatics and computational biology students reproduce current findings as part of their coursework. In one of the graduate-level courses, Intro to R Programming, we conducted a pilot study in which we asked the students to reproduce figures from published articles. The course is for students in our Master’s and PhD programs who have little to no programming experience and focus on the difficulties of analyzing real-world data. They recreated 39% out of the assigned figures, with the most significant barrier being the requirement for more sophisticated programming abilities and a lack of documentation for the methods used. We noticed that students find it fascinating and enjoyable to replicate scientific results. Properly supervised, it will allow them to make essential scientific contributions. It will also teach them about the scientific process, the relevance of reporting standards, and the value of transparency.

## Introduction

The technological advances in sequencing technologies in the last decade have led to a massive production of data and a commensurate need for biologists with programming and data-analytic skills. To help our Master’s and PhD students address this need, we developed an introductory programming course that teaches these skills. An important design decision was the programming language to use in the course. The most common ones for this field are R and Python. Both languages are open source and available on platforms running Windows, Linux/*NIX, and MacOS. For both languages, tool development is supported by large active communities that have made many libraries freely available. Python is a general-purpose programming language initially developed for software development. R was built by statisticians for statistical analysis and is widely spread in the academic community. For both, free software-development environments are available. For this course, we decided in favor of R due to *Bioconductor*, a project which provides tools and packages for the analysis of high-throughput sequencing biological data [1], giving R an advantage in the field of bioinformatics.

The typical student taking this class has no programming background and is not familiar with computer science concepts such as data structures and algorithms. Consequently, we focused more on teaching basic programming skills and less on the use of R packages for involved biological data analysis, (e.g., DEseq2, Limma, clusterProfiler). Our main objective was to familiarize the students with *R’s syntax*, *data constructs*, *conditional flow (if-else statements*, *for and while loops)*, *and functions*. The book we use for this course is *The book of R*, *A first course in Programming and Statistics* [2]. The primary materials employed are the book’s chapters and exercises, slides, and exercises created by the instructors that aim at improving the students’ understanding of various programming concepts.

On the other hand, the *reproducibility crisis* is the recognized difficulty to reproduce many scientific studies. The term was initially introduced in 2012 [3] to raise awareness of the problem resulting from unsatisfactory outcomes from large-scale reproducibility studies in the medical, biological, and behavioral sciences [4]. For example, a surprising approximately 20% to 25% reproducibility success rate was reported in Nature in 2011 in cancer research [5]. Several possible causes were conjectured to be behind the phenomenon and include HARKing [6], p-hacking [7], selective reporting of positive results [8], poor research design, or unavailability of raw data [9]. As reproducibility is a general term, there have been attempts to divide its content into more manageable terms [10]. Here, and concerning our course objectives, we focused on the term *analytic replication*, a term coined in 2016, which refers to attempts to reproduce results by reanalyzing raw data from the original publication [11].

Having the above observations in mind, we developed a teaching approach in the form of a semester project in which the students had to reproduce figures from peer-reviewed articles. Similar efforts have been reported previously by the Massachusetts Institute of Technology (MIT), Stanford University, the University of Washington, and others. These efforts focused on the field of Psychology [12,13] and Archeology [14]. To the best of our knowledge, this is the first attempt to employ the analytical replication as a teaching tool in the field of computational biology and bioinformatics.

Through this effort, our main goal was to introduce our students to real-life data analysis issues, to familiarize them with the methods used to deal with them, publication standards, and the reproductivity crisis in research. Our secondary goal was to evaluate if publications contain adequate information for a graduate-level student to reproduce part of their figures. By adequate information, we refer to any information that could be used in this reproducibility effort; for example, the data used for the analysis (raw or processed), the corresponding metadata, the list of tools and software employed accompanied with the corresponding list of parameters and the code employed. We stress that this pilot study did not concern itself with the validity of the claimed results in each of the articles we assigned to the students. In what follows, we provide more information on the project’s design and implementation and describe the students’ opinions and their stated difficulties as they navigated their projects. Lastly, we offer our conclusions on the impact that the study had on the students’ overall performance.

## Methods

In this section, we describe the design of this pilot study.

### Participants’ background and teams’ creation

Participants were students of either the Master’s or PhD programs at Thomas Jefferson University and had registered for the “Introduction to R Programming” course—a 3-credit, full-semester course. At the beginning of the semester, students provided information on their previous formal training and degrees they obtained, the program they were currently pursuing, and their programming experience, if any. We used this information to create teams of 1 or 2 people.

### Article and figure panels selection

Each team had to submit 6 scientific articles whose figures they would like to reproduce. For this selection, the students had to consider the following: (A) The papers should contain sequencing (e.g., DNAseq, RNAseq, MethylSeq, ChipSeq) or microarray data. (B) The data should be publicly available and freely accessible. (C) At least a part of the analysis presented in each article should have used the R programming language. (D) There should be at least 5 figures or figure panels in the main text or the supplementary material. To choose among each collection of candidate articles, we considered the difficulty of the article’s figures and whether they were reproducible using R.

### Goal

At the end of the project, each team had to submit their best at reproducing a minimum of 5 panels from the assigned article and the code they used for the purpose. Each team also had to document its code so that a non-expert could rerun it and regenerate the team’s results.

### Project presentations and reports

Each team members presented their assigned project in class. We provided a set of questions as a suggested guideline that they could modify according to their project’s needs. The questions formed 4 clusters: background information of the article’s field of focus; brief description of the article’s goal and findings; questions answered by each of the figures chosen for the project; and overall process questions (S1 File). The project presentations and reports had a dual purpose. First, to ensure that the students understood the paper, its experimental design, the information presented in the figures, and how this information supported its scientific findings. Second, to provide a technical report on the difficulties and obstacles the students faced in their reproducibility efforts.

## Results

### Students’ background

Six students and 1 postdoctoral fellow participated in this pilot study. The highest previously obtained degrees included Bachelor’s (4), Master’s (2), and a Doctor of Philosophy (PhD) (1), Table 1. The represented disciplines included chemistry, biochemistry, cell biology, molecular biology, and biophysics. The students’ prior programming experience ranged from none (3), to very limited (2), to moderate (2), Table *2*. Students with similar areas of expertise and similar programming experience were grouped together. This design allowed us to qualitatively measure the effect of previous programming experience on the reproducibility efforts. We notice that previous programming experience significantly affected the results of their reproducibility efforts.

**Table 1:** Students’ academic background.

Previously obtained degree	The academic level of current studies
Biochemistry Major (BS)	PhD
Biochemistry Major/Pre-Med Minor (BS)	PhD
Molecular Biophysics (BS)	Medical Doctor/PhD
Biomedical Engineering (BS), Cell Biology (MSc)	PhD
Biochemistry (BS)	PhD
Chemistry (BS and MSc)	PhD
Molecular Biology (PhD)	Postdoctoral fellow

**Table 2:** Team’s programming experience.

Team name	Number of participants	Programming experience
**Team 1**	2	No prior programming experience
**Team 2**	2	Sufficient programming experience (JAVA, MATLAB, Python, R)
**Team 3**	2	Limited programming experience
**Team 4**	1	No prior programming experience

### Selected articles and figure panels

We selected 4 articles [15–18] that appeared in journals with high-impact factors (IFs: 14.9, 14.9, 17.1, and 41.5). Because of the selection process (see Methods), the articles cover a diverse range of scientific contexts and include the human mitochondrial transcriptome [18], cancer-associated mutations of Dicer [15], regulation of the androgen receptor in prostate cancer [16], and mechanisms that establish microglial identity [17]. The total number of figure panels of each article ranged from 13 to 25. Considering the students’ programming experience and the estimated time required to reproduce each figure, we asked each team to reproduce at least 5 panels from the article’s main text or supplemental material. Teams that reported on more than 5 panels, received bonus marks for each extra panel. The selected panels included combinations of pie charts, bar plots, box plots, volcano plots, or heatmaps (S1 File, section Selected Panels).

### Students’ opinion on the projects

A summary of the students’ answers on the overall process is shown in Table *3*. Teams remained clearly motivated throughout the semester even though the project was challenging and time-consuming. All teams found that it helped them improve their R programming skills and understand well the methods that the article’s authors used to produce the figures. Also, when we asked the students what they would select if they had to choose between well-defined homework assignments or projects, they selected the latter. They considered the projects more exciting and relevant to the skill set required in their future career steps. However, they also identified the importance of both and that they complement each other. In this installment of our class, we used both the project and homework assignment, and we observed that without the latter, students would not have been able to perform the project.

**Table 3:** Answers to feedback questionnaire on the overall process of the semester’s projects.

Questions	Team 1	Team 2	Team 3	Team 4	Mean score
**Overall level of interest (1–5)**	3.5	4	5.00	5	4.38
**Overall perceived degree of difficulty (1–5)**	3.0	4	4.75	4	3.94
**In your opinion, did the project improve your R skills?**	Yes	Yes	Yes	Yes	Yes
**In your opinion, did the project improve your understanding of methods?**	Yes	Yes	NA	Yes	Yes
**Estimated hours devoted to project**	20	40	80	80	55
**Do you prefer a single project or multiple self-contained homework assignments?**	Both	Project	NA	Project	Project

### Difficulties in reproducibility

Out of the 28 panels that the students attempted to reproduce, 11 (39%) conveyed the same information as in the assigned article. By conveying the same information, we mean that the axis had the correct labeling, the bars, boxplots, pie charts, and heatmaps had the same dimensions (height, range) and similar coloring. Compare:

○ Fig 2 C, F of [18] with Fig A in S1 File panels C1, C2, F.
○ Fig 1, panels C of [17] with Fig C in S1 File panel C.
○ Fig 4, panels A and C *of* [17] *with Fig D in* S1 *File* panels A and C.
○ Fig 2, panels A, B of [15] with Fig E in S1 File panels A, B.
○ Fig 3, panels D, E and F of [15] with Fig F in S1 File panels D, E and F.
○ Supplementary Fig 6 of [15] with Fig G in S1 File.

The students had difficulty with the remaining 17 panels: For several of them, the students’ versions partially recapitulated the original, whereas the remaining panels differed completely, Table 4. To understand this outcome, we asked the students of each group to describe the difficulties that they faced and to summarize their personal experience with the datasets and the methods they used in their analyses.

**Table 4:** The number of figures reproduced.

	Team 1	Team 2	Team 3	Team 4	Total
**Total number of figures panels produced**	6	5	10	7	28
**Number of figures panels reproduced**	2	3	6	0	11

Teams used either raw or normalized data (see Methods). The data were either present on the publisher’s or journal’s website, a lab’s website, NIH’s Sequence Read Archive (SRA), or NIH’s Gene Expression Omnibus (GEO). Most teams reported that they did not face any difficulties downloading the data. However, all teams stated that the download process was time-consuming. Three of the 4 teams reported no problem with data accessibility, and 1 team reported that some of the data were either missing or not accessible. In nearly all cases, the data was well annotated. In one instance, the sample annotations used in the paper did not match exactly those in the public repository (NIH SRA) from which the data was downloaded, Table 5.

**Table 5:** Data-related answers.

Questions	Team 1	Team 2	Team 3	Team 4
**What type of data did you employ?**	Normalized and results tables	Raw SRA files	Raw and filtered	Normalized
**How did you download the data?**	Excel spreadsheets containing the results were available online.	NCBI website, Galaxy platform, R code	Links	Directly download from the GEO database
**Did you face any difficulties in downloading the data?**	No	Downloading the SRA files and converting them into Fastq format using R codes requires much more time (8–10 hours) compared to processing those data using tools on the Galaxy platform (a couple of hours).	No	No
**Did you face any difficulties in accessing the data?**	Some data was missing.	No	No	No
**Were the data well annotated?**	The excel spreadsheets were annotated very well.	Some data were not well annotated. Some samples in SRA run info use different annotations compared to the ones in the paper.	Yes	No, it is not clear how to normalize the data.

In Table 6, we summarize the students’ answers about the methods and tools they used and the overall difficulties they faced. For their work, all teams relied on the base R installation and a few selected R packages; a detailed list is available in Table 6, and instructions for their installation are in the project’s repository on GitHub. One team also employed the Galaxy platform [19]. All teams stressed that the authors did not include information about how they applied each software tool/package. Specifically, they reported that no code was made available by the authors except for 1 article [15]: but in this case, the provided code aimed to reproduce a small percentage of the analysis. The authors did not mention the parameter values used or the thresholds employed, in all cases. Because of that, it required a lot of individual work from the teams on how to implement the analysis. They had to consult the user guides for the various packages, build analysis steps from the beginning, and evaluate different combinations of parameters.

Furthermore, the initial data that a team used to start their analysis played a vital role in their difficulties. Starting the analysis from normalized or results tables made reproducibility easier than from raw data. Team 1 has a problem reproducing Fig 2E of [18] due to difficulties in identifying the parameters and metrics employed by the authors; compare Fig A in S1 File panel E with the original figure. Team 2, the most experienced team, started their analysis from raw data and employed galaxy and R to perform their analysis. As a result, Team 2’s code was more complex and required more tools and resources than the other teams. They faced difficulties identifying the correct parameters to regenerate the exact *p*-values. They failed to reproduce 3 volcano plots and a principal component analysis figure; compare Fig C in S1 File panel D with Fig 1 and Fig D in S1 File panel B with Fig 4 of [17]. Team 3 faced difficulties reproducing permutation-based *p*-values even though a part of the code was available. Due to the great stochasticity of permutation testing, the reproducibility of the *p*-values is almost impossible unless the author had “set the seed”. Setting the seed allows the same random numbers to be generated across different machines. Team 4 had several difficulties reproducing the figure and did not manage to reproduce any.

Another important point is that because our in-class lectures focused on familiarizing our students with basic R syntax and students’ lack of previous programming experience and foundations in data visualization, the instructors had several one-to-one meetings with each team. These meetings aimed to provide guidance on what R packages to employ, project organization, code examples, answer technical questions, and explain the logic behind figures’ design. Teams with less programming experience required more help versus teams with more programming experience.

Overall, previous exposure to coding and computationally involved tasks was the most critical factor in the success of reproducibility efforts. Most of the students had a degree in Biochemistry, Chemistry, and Molecular Biology, with 2 of our students having a degree in Molecular Biophysics and Biomedical Engineering, respectively (Table 1). Students with more programming experience started their analysis from the raw data (Table 5), including their effort’s tools outside the taught material (Table 6), and requiring less help. Teams with at least 1 member with previous programming experience reproduced more figures than the other teams.

**Table 6:** Method-related questions.

Questions	Team 1	Team 2	Team 3	Team 4
**What software did you use to analyze the data?**	R packages (“ggplot2,” “gplots,” “circlize”)	usegalaxy.org, R packages (“edgeR,” “statmod,” “devtools,” “ggbiplot,” “pvclust,” “gplots,” “RColorBrewer,” “bcv”)	R packages (“ggpubr,” “ggplot2,” “reshape2”)	R packages (edgeR, Glimma, org.Hs.eg.db, clusterProfiler, DOSE, TCGAbiolinks, EDASeq, ConsensusClusterPlus, ComplexHeatmap, survminer, gplots, RColorBrewer, ggplot2)
**Were the details on how to apply each software available?**	No	Details on how to apply tools in usegalaxy.org were available, and some documentation for packages was available. However, overall, it required quite a lot of individual research on how to implement the analyses.	NA	Yes, you can get the User’s Guide.
**Was there any code available to help you reproduce the results?**	No	No code was provided by the authors	The code was available. However, only 1 out of 5 codes (figures)/can be rerun with minimal effort and understanding. In addition, there was no/minimal commenting for the codes provided, making it extremely difficult to rerun or try to understand what was done to produce figures.	Packages’ user guides
**What were the difficulties that you faced?**	Our main difficulty was determining what packages to use for each figure and learning the functions.	It was time-consuming to process all the data, even with the use of tools in usegalaxy.org. Details on using certain functions and cutoff conditions in EdgeR were not available; therefore, it was challenging to generate the exact figures in the paper. The author did not publish code to process and analyze the data that could help reproduce the results.	NA	Differential Expression Analysis

## Discussion

In this paper, we described a pilot study that we used in one of our graduate-level courses. As part of the study, we asked the students to work on a semester-long project with the goal to reproduce figures from published articles. The main goal of this study was educational. We wanted to expose our students to real-world data analysis difficulties, methods used by others to tackle them, publication visualization standards, and the problem of reproducibility in research.

Importantly, the students’ feedback for the project assignments was very positive. The project’s similarity with their future work as researchers kept them motivated throughout the semester despite the difficulties they faced and the long hours. They felt that they were doing something meaningful that could possibly make a real scientific contribution. From discussions with the students, they faced several difficulties in their reproducibility efforts. For example, changing program parameters could generate different pictures leading to different scientific conclusions. Due to this, they understood the importance of transparency and standards in scientific reporting, which in that case would translate into code sharing to the minimum. They understood the importance of standards and openness in scientific reporting. On the technical aspect of things, all the students viewed this approach as a good way to hone their R programming skills and improve their understanding of how to analyze biological data. We also touched upon best practices to make their analysis more reproducible. We asked them to document and share the code they used to generate their figures so that a non-expert could reproduce their work. The code is available on the project’s page on GitHub. We say touch upon as there are available tools and platforms to support reproducibility efforts such as GitHub, docker, and codeocean. However, these are for experienced programmers and unsuitable for novices. We asked them to provide the code they used as the minimum that we consider a part of best practice but suitable for the level of our students.

Regarding difficulties, all students reported similar experiences. There were 2 recurrent observations. First, nearly all groups reported that no code had been made available by the authors or their assigned article. This forced them to spend a notable amount of time in data procurement, data processing, and data analysis, which may have contributed to the inability to reproduce some of the figures. Second, all groups reported the absence of a detailed explanation of how each analytical method was applied. The groups overcame this difficulty by relying on the documentation and user guides available for each package and by trying out different parameter combinations to “guess” the ones used by the articles’ authors. Furthermore, in cases where some code was provided, it lacked documentation and rerunning it generated some differences from the published results, which could lead to different scientific conclusions. We believe that this trial-and-error process was one factor that made the projects so time-consuming and resulted in only 11 out of 28 (39%) figure panels conveying the same information.

The difficulties that our students reported supported previously identified difficulties in *analytic replication* [11]. An alternative term, *Methods reproducibility*, refers to the same problem but has a broader meaning [10], quoting from Ioannidis’ paper, *“refers to the provision of enough detail about study procedures and data so the same procedures could*, *in theory or in actuality*, *be exactly repeated*.*”* It is worth noting that the scientific community widely accepts the problem of reproducibility in research. In the survey published in Nature in 2016, 70% of researchers have tried and failed to reproduce another scientist’s experiments, and 52% of those surveyed agree there is a significant reproducibility crisis [20]. However, we would like to accentuate that we do not suggest any concrete evidence of the reproducibility crisis due to this approach’s experimental setting and limitations. Our students limited to no programming experience was essential in reproducing only a limited number of figures. In fact, we observed that students with more programming experience could reproduce more figures. Also, this was a semester-long project, and we believe that had the students been given more time, they would have been able to recreate more figures.

Furthermore, several lessons were learned from this effort. (A) These projects would have been more appropriate for students with advanced programming skills. (B) Being part of classes that focus on RNA or DNA sequencing data analysis would have been more suitable. (C) Another consideration is the number of members a team should include to reproduce a published work. In our case, the teams consisted of up to 2 members and were assigned 5 figures per team. However, these figures amount to a small percentage of the total number per article (13 to 25 in total). Assigning more or all figures would have given us a better understanding of whether the publication is reproducible. Teams with more members or more time could have solved that issue. (D) Also, for not experienced students in programming, well-defined homework assignments must be present in the curriculum to allow the students to understand and implement basic concepts. In this installment of our class, we used both the project and homework assignment, and we observed that without the latter, students would not have been able to perform the project.

In conclusion, this teaching approach could benefit the students and the general scientific community. The gold standard for verifying the credibility of published scientific findings is replication. We observed in our classes that students replicating state-of-the-art scientific findings are stimulating and fun. With proper supervision, instructors must ensure that students’ efforts are correct and appropriately done; it will allow students to make essential scientific contributions and become a driving force in efforts to solve the reproducibility crisis. It will also provide practical lessons about the scientific process, the significance of reporting standards, and the importance of openness. Through this study, we want to invite other institutions to join forces to tackle the reproducibility crisis by utilizing their courses and students. We believe every institution with the appropriate curriculum and equipment could perform a reproducibility analysis. Above all, such an effort will help the students to sharpen their skills by solving real-life data analysis problems. However, students should be mature enough and have the proper experience. We noticed that inexperienced students faced several difficulties due to a lack of programming experience. Lastly, we propose that students should get exposed to the reproducibility crisis problem to understand its importance and be taught the tools and resources available to solve it.

## Supporting information

S1 File Supplementary material of reproducibility efforts as a teaching tool: A pilot study. S1 File includes the questions provided as a suggested guideline and the figures generated by the teams. Fig A in S1 File: Reproducing figure 2, panels A, B, C, E, and F from the original publication (1). Panels C and F were reproduced. Fig B in S1 File: Reproducing Fig 5A of the initial publication (1). The figure was not reproduced. Fig C in S1 File: Reproducing Fig 1, panels C and D of the initial publication (2). Panel C was reproduced. Fig D in S1 File: Reproducing Fig 4, panels A, B, and C of the initial publication (2). Panels A and C were reproduced. Fig E in S1 File: Reproducing Fig 2, panels A, B, and C of the initial publication (3). Panels A and B were reproduced. Fig F in S1 File: Reproducing Fig 3, panels D, E, and F of the initial publication (3). All panels were reproduced. Fig G in S1 File: Reproducing Supplementary Fig 6 of the initial publication (3). The figure was reproduced. Fig H in S1 File: Reproducing Fig 4 panels A and B of the initial publication (4). The figure was not reproduced. Fig I in S1 File: Reproducing Fig 4 panel C of the initial publication (4). The figure panel was not reproduced.
(DOCX)
