# Supplementary material for: Reproducibility efforts as a teaching tool: A pilot study
Source: PLoS Comput Biol. 2022 Nov 10;18(11):e1010615. doi: 10.1371/journal.pcbi.1010615 (PMC9648701; doi:10.1371/journal.pcbi.1010615)
Supplement: S1 File — includes the questions provided as a suggested guideline and the figures generated by the teams. Fig A in S1 File: Reproducing figure 2, panels A, B, C, E, and F from the original publication (1). Panels C and F were reproduced. Fig B in S1 File: Reproducing Fig 5A of the initial publication (1). The figure was not reproduced. Fig C in S1 File: Reproducing Fig 1, panels C and D of the initial publication (2). Panel C was reproduced. Fig D in S1 File: Reproducing Fig 4, panels A, B, and C of the initial publication (2). Panels A and C were reproduced. Fig E in S1 File: Reproducing Fig 2, panels A, B, and C of the initial publication (3). Panels A and B were reproduced. Fig F in S1 File: Reproducing Fig 3, panels D, E, and F of the initial publication (3). All panels were reproduced. Fig G in S1 File: Reproducing Supplementary Fig 6 of the initial publication (3). The figure was reproduced. Fig H in S1 File: Reproducing Fig 4 panels A and B of the initial publication (4). The figure was not reproduced. Fig I in S1 File: Reproducing Fig 4 panel C of the initial publication (4). The figure panel was not reproduced. (DOCX) [file pcbi.1010615.s001.docx]

Supplementary Material of

Reproducibility Efforts as a Teaching Tool: A Pilot Study

# Questions provided as a suggested guideline

## Students’ background information

- Students’ previously obtained degrees (Bachelor / Master).
- Students’ programming experience before the introduction to R programming class.
- The Thomas Jefferson University program they were attending.

## Brief paper description

- What is the general scientific question of the paper?
- What type of data were produced and how they were utilized to support the scientific findings?

## Specific figures questions

1. Describe the figures you tried to reproduce. What information were they trying to convey?
2. Describe how you reproduced the figure.
3. Data related Questions
   1. What type of data did you employ? (Raw, normalized, Results tables)
   2. How did you download the data?
   3. Did you face any difficulties in downloading the data?
   4. Did you face any difficulties in accessing the data?
   5. Were the data well annotated?
4. Method related Questions
   1. What software did you use to analyze the data? (R libraries and other type of software)
   2. Were the details on how to apply each software available?
   3. Was there any code available to help you reproduce the results?
   4. What were the difficulties that you faced?
5. Compare your figure to the original.

## Overall Process Questions

1. Evaluate the overall difficulty of the semester’s project from 1 to 5. 1 being very easy, 5 being very difficult. Explain your answer.
2. How much time did you spend in hours to complete the project?
3. How many figures did you manage to reproduce?
4. Did you find the semester project interesting? 1 being not interesting, 5 being very interesting. Explain your answer.
5. Did you find this project useful in improving your R programming skills?
6. Did you find this project useful in understanding methods employed in the analysis of biological data?
7. If you had to choose between this type of project versus well-defined in class assignments, which one would you choose and why?

# Selected Panels

## Team 1

Team1 tried to reproduce figure2 panels A, B, C, E, F, and figure 5 panel A from publication (1).


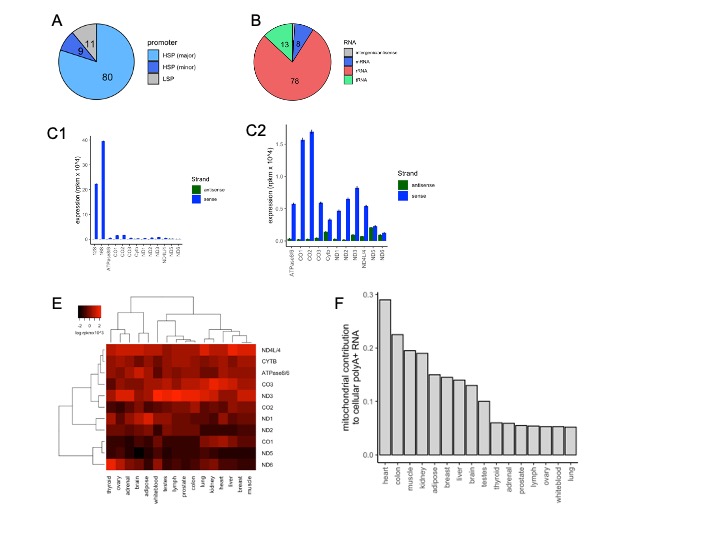
 **Fig A.** Reproducing figure 2, panels A, B, C, E and F from the original publication (1). Panels C and F were reproduced.


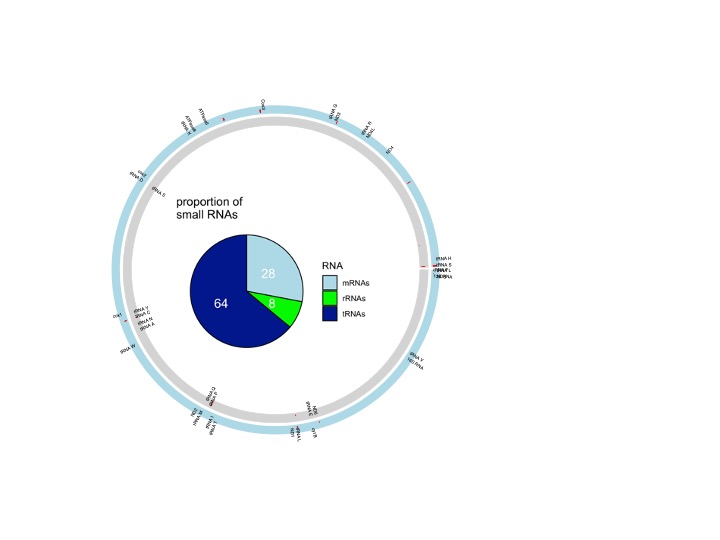


**Fig B.** Reproducing Figure 5A of the initial publication (1). The figure was not reproduced.

## Team 2

Team2 tried to reproduce figure1 panels C, D and figure 4, panels A, B and C from publication (2).


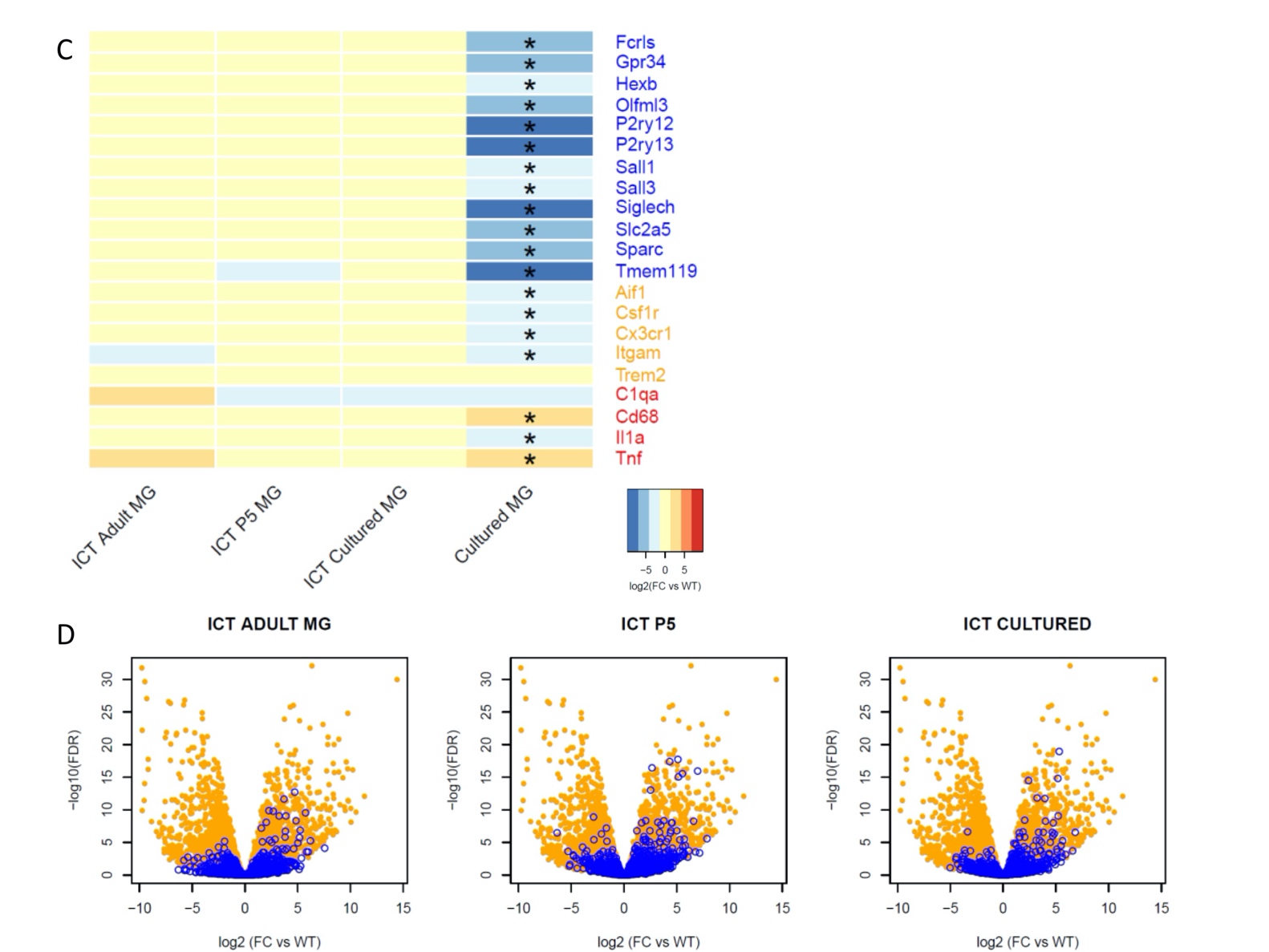


**Fig C.** Reproducing Figure 1, panels C and D of the initial publication (2). Panel C was reproduced.


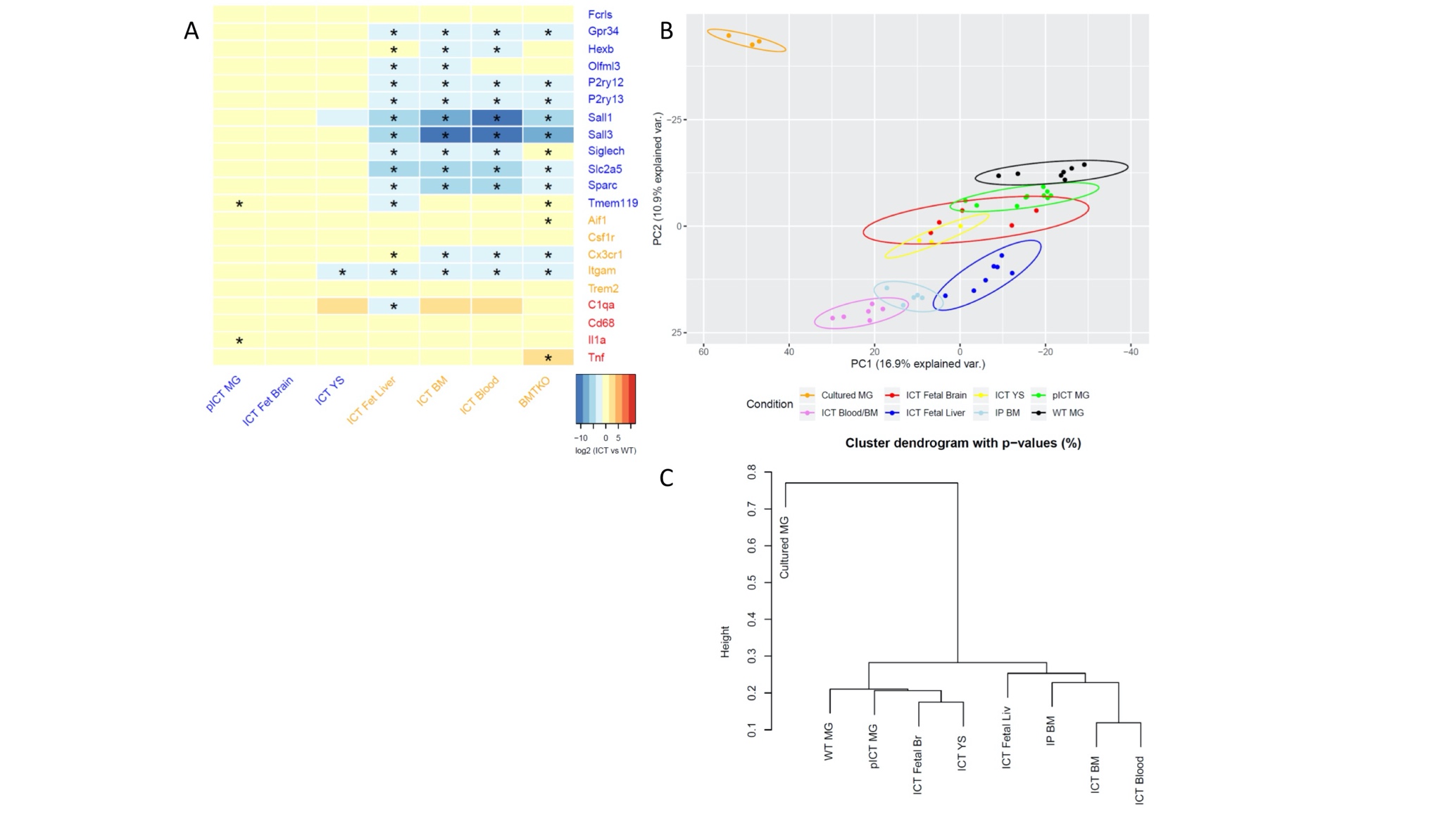


**Fig D.** Reproducing Figure 4, panels A, B and C of the initial publication (2). Panels A and C were reproduced.

## Team 3

Team 3 tried to reproduce figure2 panels A, B, C; figure 3, panels D, E, F; supplementary figure 2; supplementary figure 3 panels A and B, and supplementary figure 6 from publication (3).


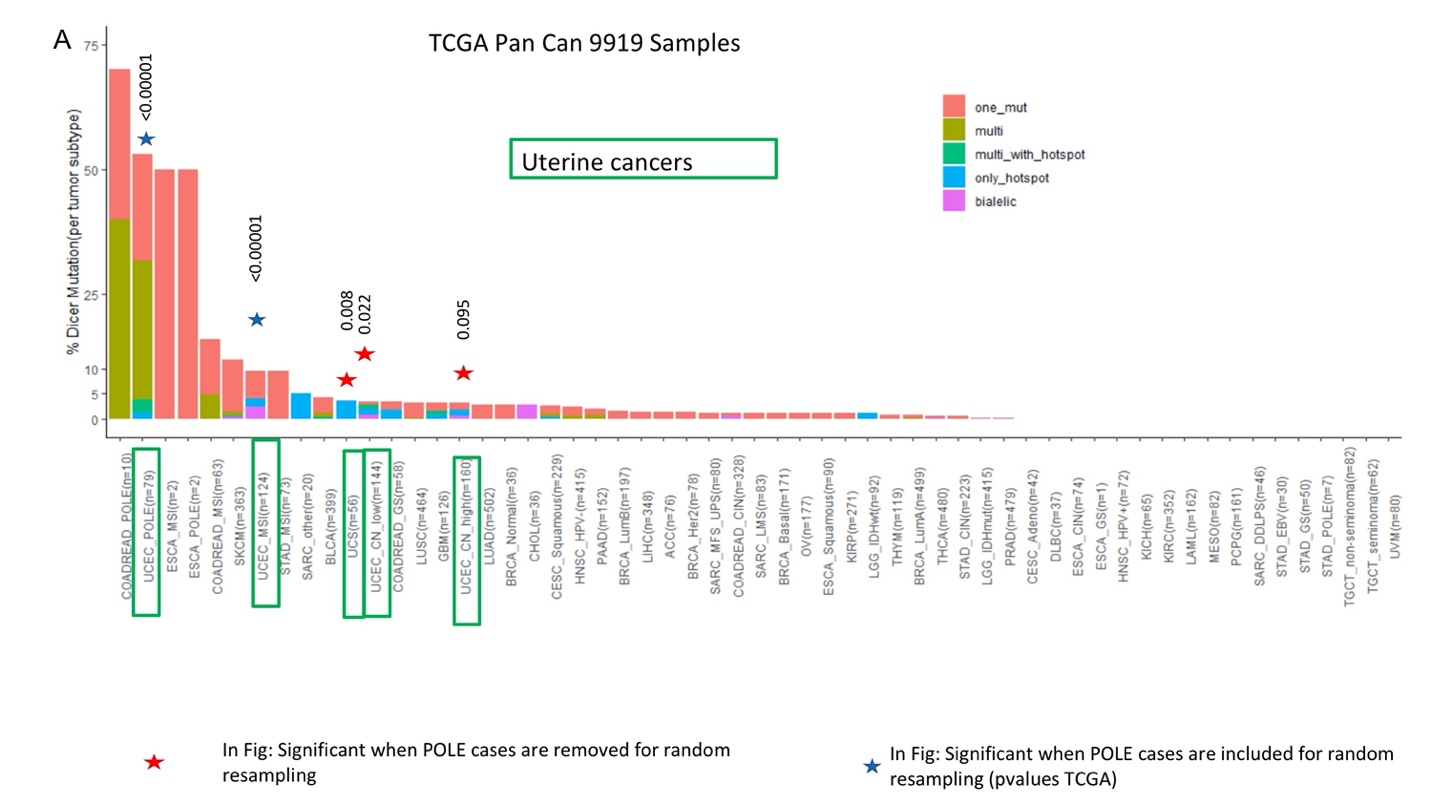


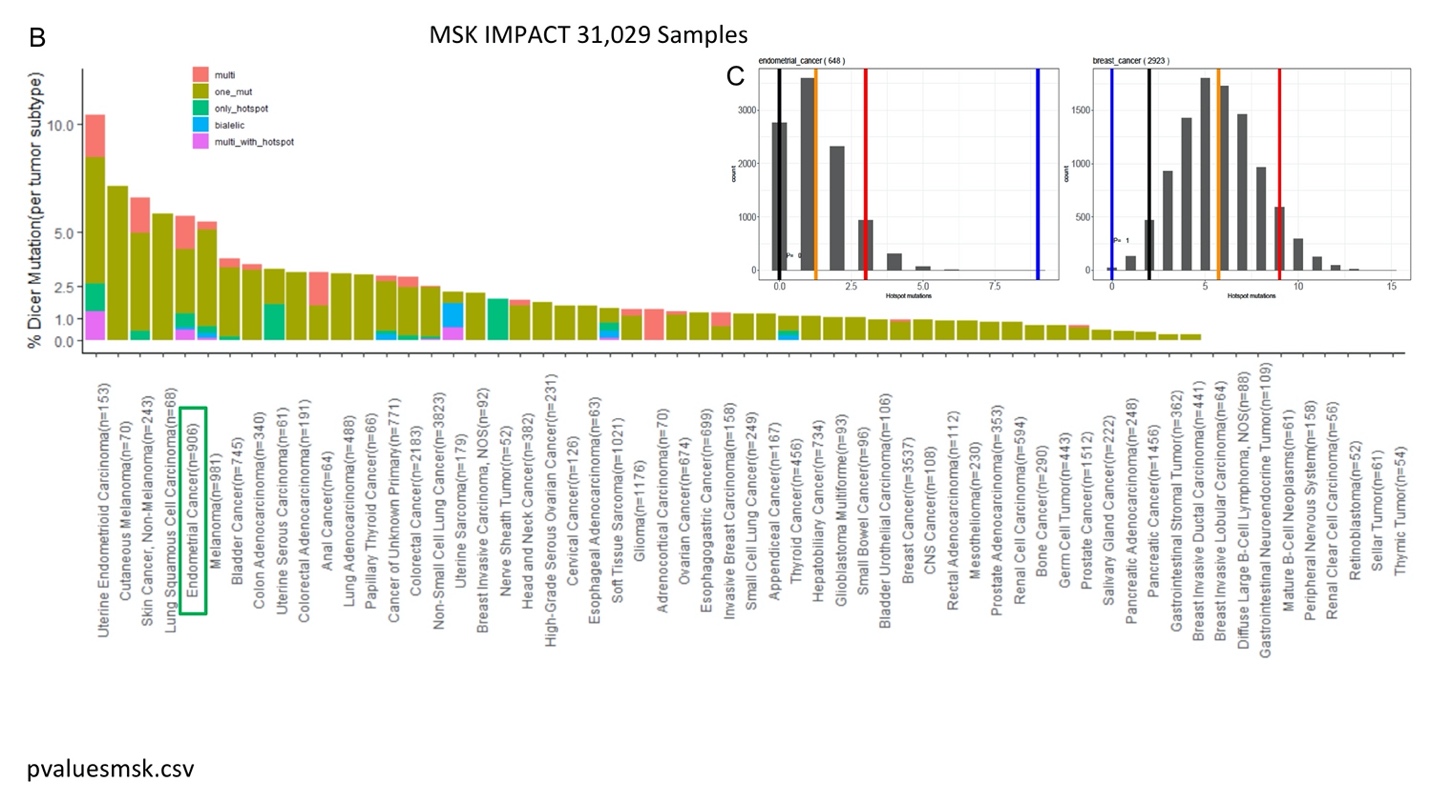


**Fig E.** Reproducing Figure 2, panels A, B and C of the initial publication (3). Panels A and B were reproduced.


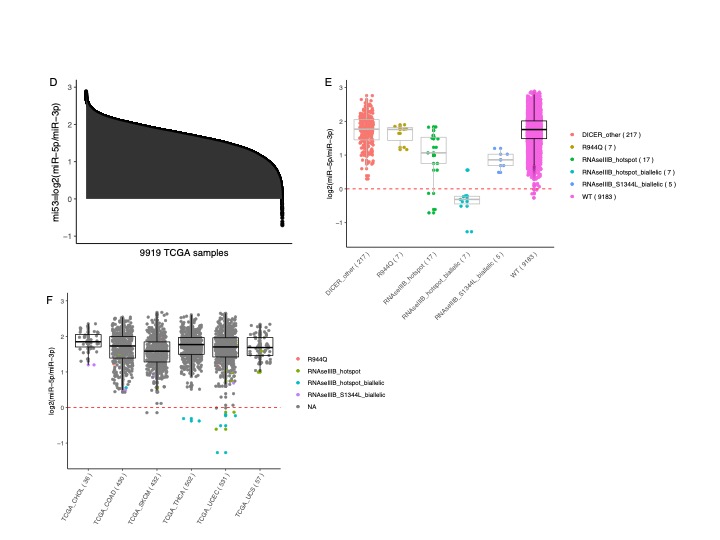


**Fig F.** Reproducing Figure 3, panels D, E and F of the initial publication (3). All panels were reproduced.


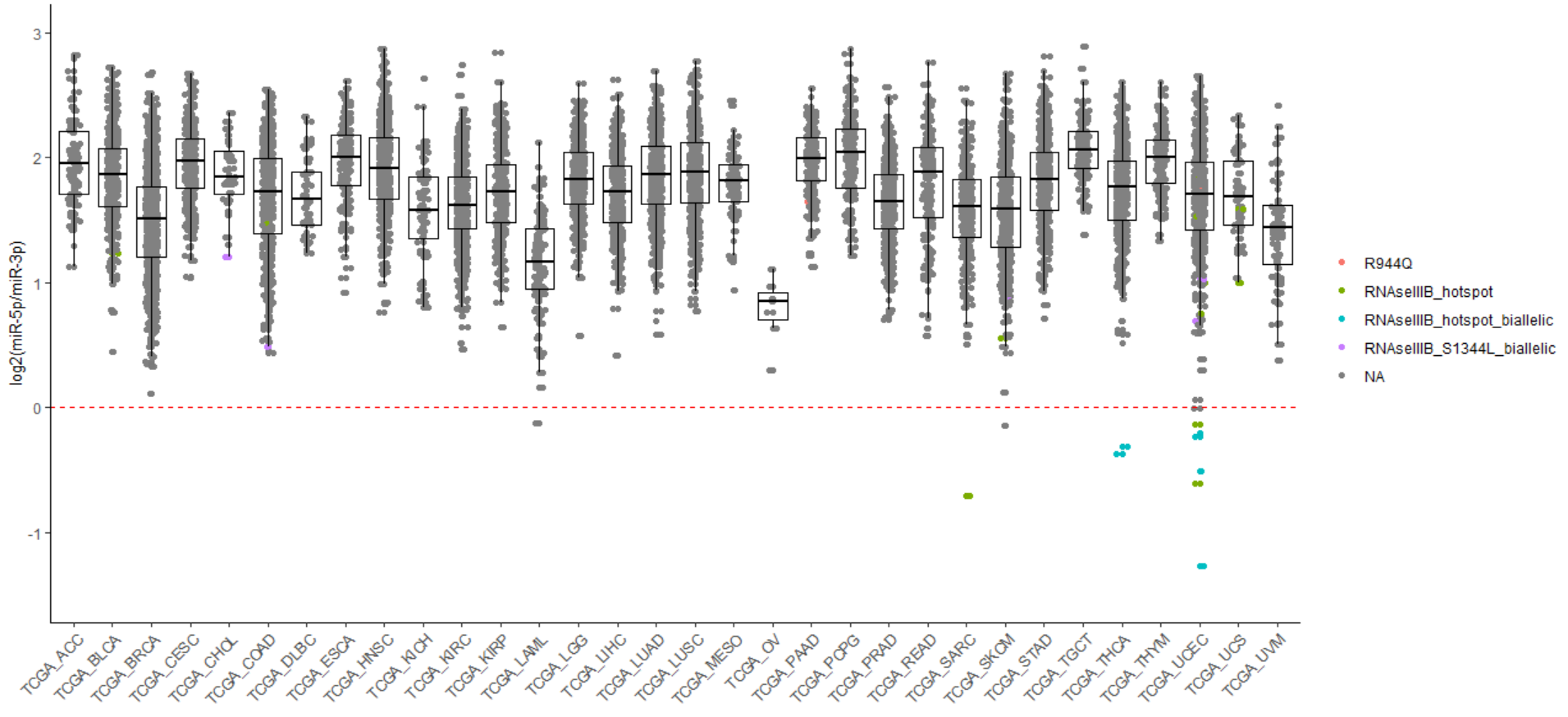


**Fig G.** Reproducing Supplementary Figure 6 of the initial publication (3). The figure was reproduced.

## Team 4

Team 4 tried to reproduce figure 4 panels a, b and c from publication (4).

No figures were reproduced.


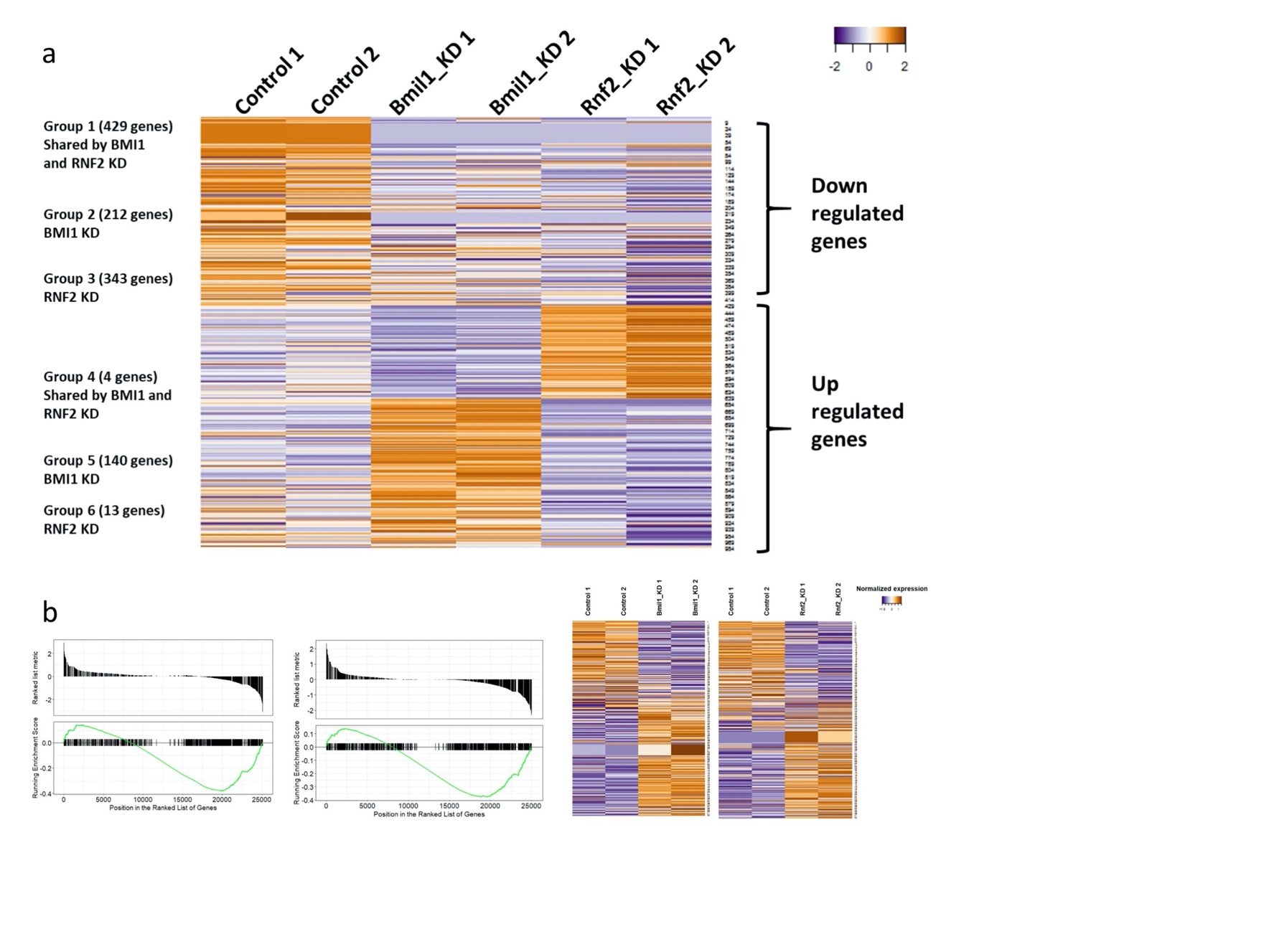


**Fig H**. Reproducing Figure 4 panels a and b of the initial publication (4). The figure was not reproduced.


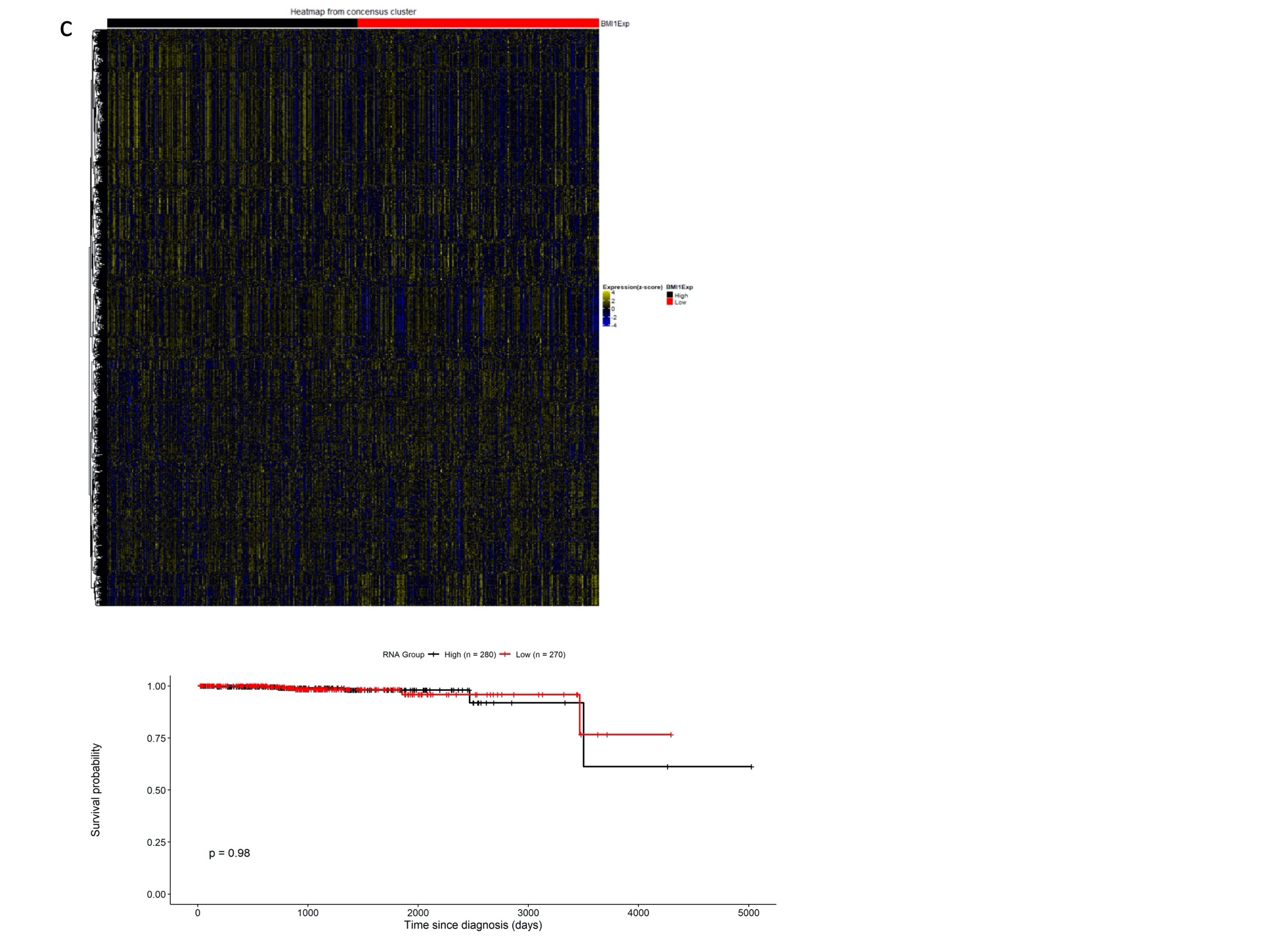


**Fig I.** Reproducing Figure 4 panel C of the initial publication (4). The figure panel was not reproduced.

# References

1. Mercer TR, Neph S, Dinger ME, Crawford J, Smith MA, Shearwood AMJ, et al. The human mitochondrial transcriptome. Cell [Internet]. 2011;146(4):645–58. Available from: http://dx.doi.org/10.1016/j.cell.2011.06.051

2. Bennett FC, Bennett ML, Yaqoob F, Mulinyawe SB, Grant GA, Hayden Gephart M, et al. A Combination of Ontogeny and CNS Environment Establishes Microglial Identity. Neuron [Internet]. 2018;98(6):1170-1183.e8. Available from: https://doi.org/10.1016/j.neuron.2018.05.014

3. Vedanayagam J, Chatila WK, Aksoy BA, Majumdar S, Skanderup AJ, Demir E, et al. Cancer-associated mutations in DICER1 RNase IIIa and IIIb domains exert similar effects on miRNA biogenesis. Nat Commun [Internet]. 2019;10(1). Available from: http://dx.doi.org/10.1038/s41467-019-11610-1

4. Zhu S, Zhao D, Yan L, Jiang W, Kim JS, Gu B, et al. BMI1 regulates androgen receptor in prostate cancer independently of the polycomb repressive complex 1. Nat Commun [Internet]. 2018;9(1):1–13. Available from: http://dx.doi.org/10.1038/s41467-018-02863-3
